# Stress-Induced Hyperprolactinemia: Pathophysiology and Clinical Approach

**DOI:** 10.1155/2018/9253083

**Published:** 2018-12-03

**Authors:** Samara Levine, Ozgul Muneyyirci-Delale

**Affiliations:** Department of Obstetrics and Gynecology, SUNY Downstate Medical Center, Brooklyn, NY, USA

## Abstract

While prolactin is most well known for its role in lactation and suppression of reproduction, its physiological functions are quite diverse. There are many etiologies of hyperprolactinemia, including physiologic as well as pathologic causes. Physiologic causes include pregnancy, lactation, sleep-associated, nipple stimulation and sexual orgasm, chest wall stimulation, or trauma. Stress is also an important physiologic cause of hyperprolactinemia, and its clinical significance is still being explored. This review will provide an overview of prolactin physiology, the role of stress in prolactin secretion, as well as the general clinical approach to hyperprolactinemia.

## 1. Introduction

### 1.1. Prolactin Physiology

#### 1.1.1. Structure

Prolactin is a peptide containing 198 amino acids; it shares genetic, structural, and binding properties with growth hormone and human placental lactogen [1]. The prolactin gene is encoded on chromosome 6. In circulation, prolactin exists in different forms: a monomeric form whose molecular weight (MW) is 22 kDA, a polymeric form, “big prolactin” whose MW is 50–60 kDa, and a larger polymeric form, “macroprolactin,” whose MW is greater than 100 kDa. The smallest form is biologically active and accounts for 80–95% of prolactin found in circulation. Of note, big prolactin and especially macroprolactin, due to their large size, have increased clearance time however do not contribute to hyperprolactinemia symptoms due to their biologic inactivity [2, 3].

#### 1.1.2. Metabolism

Normal adult serum prolactin levels vary between sexes: for women between 10 and 25 *µ*g/L and 10–20 *µ*g/L in men. Like many hormones, prolactin secretion is pulsatile, with maximum secretion during REM sleep, with typical peak between 4 and 6 AM [1]. Prolactin is eliminated by the liver and the kidney, and the half life of circulating prolactin is 20–50 minutes [1, 2].

#### 1.1.3. Secretion

Lactotrophs, representing about 20% of the cells encompassing the anterior pituitary and found in its lateral portion, secrete prolactin [2]. Control of prolactin secretion is primarily inhibitory, with dopamine suppressing prolactin release, specifically through the pituitary dopamine type 2 (D2) receptors [1]. Prolactin is also synthesized in the decidualized stroma of endometrial tissue, however, this prolactin is unresponsive to dopamine [2]. On the contrary, thyroid-releasing hormone (TRH) elicits prolactin release, with an effect seen within 15–30 minutes of IV infusion [1]. Estrogen mediates this by enhancing the effect of TRH and inhibiting that of dopamine. Vasoactive intestinal peptide, oxytocin, galanin, and PHM-27 induce prolactin release, as does serotonin, which is thought to be the major stimulating factor accounting for the rise of prolactin during REM sleep. Conversely, glucocorticoids, thyroid hormone, GABA, and somatostatin weakly suppress prolactin secretion [1, 4, 5]. Additionally, cytokines IL-1, IL-2, and IL-6 stimulate prolactin secretion, while INFγ and endothelin-3 are inhibitory cytokines [6].

#### 1.1.3. Function

Along with GH and IL-6 receptors, prolactin receptors are a member of the type I cytokine receptor family. Prolactin is key for induction and maintenance of lactation. It inhibits reproductive function through suppression of GnRH, thus suppressing the release of gonadotropins FSH and LH and impairing gonadal steroidogenesis in women and men [1]. It is important to note that prolactin also plays a role in growth and development, water and electrolyte balance, metabolism, and immunoregulation [7]. Of note, prolactin has been identified to play a vital role in hepatocyte turnover and growth [8], increase intestinal mucosa [9], induce proliferation of vascular smooth muscle [10], as well the B cells of the pancreas [11]. Prolactin stimulates T and B cells, natural killer cells, macrophages, neutrophils, CD34+ hematopoietic cells, and antigen presenting dendritic cells [12]. Additionally, prolactin is produced by adipose tissue and stimulates adipogenesis and inhibits lipolysis; it promotes insulin sensitivity [13].

### 1.2. Hyperprolactinemia

#### 1.2.1. Epidemiology

Hyperprolactinemia is the most common pituitary hormone hypersecretion syndrome in both men and women [14]; it most commonly affects women aged 25–34 years old and male occurrence is 4 times less frequent [15]. Prevalence in the general population is approximately 0.4%, estimated to affect between 10 and 90 people per 100,000 [15, 16]. However, hyperprolactinemia is present in 15–20% of women presenting with menstrual disturbances [2].

#### 1.2.2. Presentation

Clinical presentation of hyperprolactinemia is sex-specific. Women often present with abnormal menstruation, including irregular cycles, amenorrhea, oligomenorrhea, hypomenorrhea, hypermenorrhea, or shortened menstrual cycles (polymenorrhea). Additional findings may include galactorrhea, infertility, decreased libido, dyspareunia, acne, weight gain, increased adiposity, and hirsutism [13–15]. Hyperprolactinemia causes abnormal frequency and amplitude of female LH pulsations, perhaps through interference with positive estrogen effect on midcycle LH release. There is evidence that hyperprolactinemia inhibits gonadotropin release rather than synthesis. Additionally, elevated prolactin directly inhibits basal and gonadotropin-stimulated ovarian secretion of estradiol and progesterone [2]. In women, chronic hyperprolactinemia causes decreased bone mineral density, especially in the setting of significant hypoestrogenism. In men, hyperprolactinemia may present acutely with diminished libido and infertility. Chronic hyperprolactinemia may present with osteopenia, reduced muscle mass, and decreased beard growth [14].

#### 1.2.3. Diagnosis

Clues in patient's history as well as physical exam may suggest hyperprolactinemia, especially in the setting of evaluation for menstrual disturbances or infertility. Important historical findings include secondary amenorrhea, decreased libido, and galactorrhea. On physical exam, providers should assess mammary glands, skin, in particular looking for presence of acne or hair growth, as well as neurological exam, specifically assessing visual fields. Diagnosis is confirmed with a blood test. Optimal timing of blood test is 2–3 hours after waking. Hyperprolactinemia is diagnosed when blood prolactin concentration is greater than 25 ng/ml. An elevated prolactin level, especially one that is mildly elevated (20–40 ng/ml), should be confirmed with at least two tests to account for circadian fluctuation as well as other environmental factors causing transient elevation; however, it can be diagnosed with a single value if the level exceeds the upper limit of normal by at least five-fold [3, 15]. The hyperprolactinemia rest test, with serial blood sampling from an indwelling catheter as a patient rests in a quiet room, found that samples drawn at 0, 30, and 60 minutes, were sufficient to diagnose stress-induced hyperprolactinemia, with mild to moderate elevations in prolactin, between 20 and 100 ng/mL [17]. Another study found that intravenous catheterization with 15-minute rest period did not correct elevated prolactin, which is as expected with the known half life of prolactin [18].

In reproductive age women, it is important to check pregnancy status, as prolactin levels can increase 10-fold in pregnancy, reaching 300 ng/ml in some individuals. Additionally, the macromolecule prolactin, which is not biologically active, has increased clearance time and may contribute to the findings of elevated prolactin levels, without the clinical relevance. If this is suspected, diagnosis of macroprolactinemia can be made by applying polyethylene glycol to patient's serum before performing the assay, to precipitate the macroprolactin [3]. Etiologies of hyperprolactinemia are listed in Table 1, and pharmacologic agents affecting prolactin levels are discussed in Table 2.

### 1.3. Prolactin and Stress Response

The physiologic response to stress is complex; it includes norepinephrine release from various parts of the CNS, secretion of epinephrine from the adrenal medulla, as well as hypothalamic-pituitary-adrenal (HPA) axis activation. Stress response also includes the release of prolactin, which is of significant clinical relevance as there is substantial evidence that prolactin plays a significant role in the development of stress-induced pathology, including stress-induced intestinal epithelial barrier dysfunction [25], stress-induced tracheal epithelial barrier dysfunction [26], cardiac dysfunction in peripartum cardiomyopathy [27], and psychological stress in the development of cardiovascular pathology [28]. Further understanding the role of prolactin in modulating emotional stress is essential and can provide further insight into systemic conditions arising secondary to hyperprolactinemia.

#### 1.3.1. Stress-Induced Hyperprolactinemia: Physiology

Prolactin receptor expression in the adrenal cortex of several species supports an evolutionary role of prolactin in the stress response [29]. There is a plethora of evidence supporting prolactin's role in the adrenal gland's response to stress, including that hyperprolactinemia increases secretion of ACTH [2, 30, 31], induces adrenal hypertrophy, and increases storage of cholesterol esters [29]. Moreover, it is thought that hyperprolactinemia increases the adrenal cortex's sensitivity to ACTH, thus resulting in high corticosterone release even in the setting of low levels of ACTH [2]. Prolactin may also directly induce adrenal steroidogenesis; prolactin has been found to increase levels of adrenal androgens, dihydroepiandrosterone and dihydroepiandrosterone sulfate [32], cortisol and aldosterone [33] and stimulate adrenal catecholamine synthesis [7]. Prolactin acts via G-protein adenylate cyclase coupling and cyclic AMP production [34]; it is hypothesized that prolactin-induced corticosterone release is mediated by increased cAMP production [2], as well as via 3B-hydroxysteroid dehydrogenase activity [34]. The role of prolactin in modulating adrenal innervation is controversial [2].

*Neuroendocrine Changes.* There is some evidence that functional hyperprolactinemia may be due to stress-induced neuroendocrine changes of dopamine and serotonin, thus affecting prolactin release [35]. REM sleep rebound, a behavioral coping mechanism to stress, has been found to be mediated by prolactin [36]. As noted earlier, peaks in the prolactin during sleeping are thought to be mediated by serotonin, thus suggesting complex responses to stress, which requires further research to explore and is beyond the scope of this review.

*Maintaining Homeostasis.* Some studies suggest that prolactin secretion during stress acts to maintain homeostasis within the immune system. While glucocorticoids, also released in response to stress, are suppressive immunomodulators, prolactin and growth hormone stimulate the immune system [12]. In vivo studies support a protective role of prolactin after trauma-induced hemorrhage, burn injury, infection, and glucocorticoid administration, with prolactin preventing glucocorticoid-induced lymphocyte cell death, as well as improving macrophage and splenocyte function and antagonizing the immunosuppressive effects of TGF-B, TNF-a, and corticosterone [12]. Prolactin may also play an important role in maintaining metabolic homeostasis. In male rats, prolactin levels of 60–80 ng/mL, levels consistent with those seen as a response to stress, increased circulating adiponectin and reduced expression of proinflammatory mediators to improve insulin sensitivity and decrease adipose tissue dysfunction [13].

#### 1.3.2. Types of Stress

Considerable prolactin secretion occurs when animals are exposed to physical or psychological stress [37]. In male mice, 15 minute period of restraint stress causes a 7-fold increase in circulating prolactin levels [38]. In these same mice, this stress-induced increase in prolactin was shown to interact with peripheral targets, significantly increasing tyrosine-phosphorylated signal transducer and activator of transcription 5 (pSTAT5) in the arcuate nucleus, median eminence, and zona fasiculata of the adrenal cortex, as well as central targets, reducing pSTAT5 staining of tuberoinfundibular dopaminergic neurons [38]. In mice, prolactin infusion into cerebral ventricles prevented formation of stress-induced gastric ulcers as well as had antidepressant effects during forced swimming [12]. There is evidence that prolactin also mediates pathological effects of chronic stress. Chronic stress induces expression of prolactin receptors in choroid plexus cells [12] and upregulates prolactin receptor expression in the heart [28]. In mice models, stress-derived prolactin generated fibrofatty cells in the heart, which was prevented with administration of prolactin antagonists [28]. This finding suggests a further complexity regarding prolactin's role in acute vs chronic stress, where acutely it may produce a proinflammatory state, which is protective, but chronic exposure to heads to habituation to prolactin and pathology may ensue.

Psychology research has found hyperprolactinemia to be more common in individuals with mental illness than the general population [39]. It is hypothesized that the increase in dopamine in psychosis may be, in part, a regulatory response to down regulate the stress-induced rise in prolactin [40]. There is evidence that situations associated with passive coping cause an increase in prolactin, however situations of active coping do not affect prolactin [35]. Additionally, childhood exposure to an absent, alcoholic, or violent father may predispose women to developing hyperprolactinemia later in life [41]. Similarly, studies conducted under hypnosis found prolactin secretion increased with anger from humiliating experiences, while cortisol was associated with surprise and intimidation. This finding supports a complex neuroendocrine response to emotional distress that is unjustly simplified and labeled: it is of clinical importance for providers to more pointedly identify stressors and emotions. Psychology research has hypothesized the role of prolactin as a biomarker and regulator of “maternal subroutine,” along with other important hormones, with the goal of optimizing mother-child relationships and as such, has complex relationship with environmental stimuli [42].

#### 1.3.3. Approach

While more research is necessary to guide clinical practice, it is suggested that stress related to seeing the physician can cause elevation in prolactin. It is suggested that if the prolactin levels are less than 50 ng/ml, blood sample should be redrawn after the patient has rested in a quiet room for 60 minutes [2, 17].

### 1.4. Treatment

Approach to treatment requires assessment of severity of symptoms, desire to reproduce, and evaluation of underlying cause. The mainstay for treatment of symptomatic hyperprolactinemia, particularly patient's with anovulation, infertility, or galactorrhea, is dopamine agonists, specifically bromocriptine and carbergoline. Bromocriptine has a short half life and requires daily (at bedtime) or twice daily dosing and is often associated with gastrointestinal side effects. Carbergoline, a selective D2 receptor agonist, has fewer side effects, greater potency, and longer half life, compared to bromocriptine, and can be dosed biweekly. At high doses (>3 mg daily), however, carbergoline has been associated with hypertrophic valvular heart disease and chronic use at low doses may increase risk of valvular heart disease. If individuals are unable to tolerate oral agents, vaginal agents are available, with fewer side effects. Both bromocriptine and carbergoline are considered safe in early pregnancy and thus can be used in women of reproductive age or those considering conceiving [3].

Alternative treatments are also available, including cyclic progestin, OCPs, or estrogen replacement therapy [2, 3]. Additionally, there is evidence that adjunctive metformin significantly lowers prolactin levels and alleviates symptoms of hyperprolactinemia as compared to bromocriptine therapy alone [43]. Of note, metformin therapy, when combined with low-dose bromocriptine, was found to improve glucose and lipid homeostasis [44].

### 1.5. Further Discussion/Future Direction

Prolactin is a unique hormone in its wide reaching effects as well as its modulation by physical, psychological, and environmental factors. While this review provides an overview on the current understanding of the relationship between stress and prolactin, further research is needed to further elucidate the mechanisms of stress-induced prolactin secretion as well as the implications in pathological conditions. In the field of psychiatry, there is increasing interest in the potential role of prolactin as a biological marker of suicidal activity, specifically whether raised postmortem prolactin levels can detect antemortem physical stress [45]. Additionally, further studies are needed to confirm or refute prolactin's role in the development of psychosis [16]. In regards to hyperprolactinemia in emerging psychosis, women have been found to have higher prolactin levels, even after correcting for biological variation, which may suggest a sex-dependent stress response with prolactin, which should be further elucidated [12]. Moreover, additional research is needed to further elucidate prolactin's regulatory role in metabolic homeostasis and immune system function.

## Figures and Tables

**Table 1:** Common etiologies of hyperprolactinemia [2, 5, 6, 14, 15, 19, 20].

Physiologic causes	Pregnancy, nipple stimulation, stress, lactation, sexual intercourse/Sexual orgasm, venipuncture, chest wall stimulation (trauma, herpes zoster), high protein diet, exercise, hypoglycemia
Pituitary disease	Prolactinomas (microadenomas and macroadenomas); acromegaly, empty sella syndrome; lymphocyctic hypophysitis
Hypothalamic disease	Craniopharyngiomas, meningiomas, dysgerminomas, Rathke's pocket cyst, other tumors, sarcoidosis, eosinophilic granuloma, neuraxis irradiation, arteriovenous malformations, pituitary stalk section
Neurogenic	Chest wall lesions, spinal cord lesions
Systemic disease	Hypothyroidism, chronic renal failure, hepatic cirrhosis, Cushing's disease; Addison's disease, histiocytosis X, temporal arteries inflammation; chronic uremia; SLE; multiple sclerosis; Sjogren's syndrome
Other	Pseudocyesis, polycystic ovary syndrome; epilepsy; meningitis, mutation in prolactin receptor gene (His188Arg)

**Table 2:** Pharmacologic agents affecting prolactin concentrations [21–24].

*Stimulators*
Anesthetics, including cocaine
Antipsychotics 1^st^ generation (chlorpromazine^*∗∗∗*^, fluphenazine^*∗∗∗∗*^, haloperidol^*∗∗∗∗*^, loxapine^*∗∗∗*^, perphenazine^*∗∗∗*^, pimozide^*∗∗∗*^, thiothixene^*∗∗∗*^, trifluoperazine^*∗∗∗*^)
Antipsychotics, 2^nd^ generation (aripiprazole^*∗*^, asenapine^*∗∗∗*^, clozapine^*∗*^, iloperidone^*∗*^, lurasidone^*∗*^, olanzapine^*∗∗*^, paliperidone^*∗∗∗∗*^, quetiapine^*∗*^, risperidone^*∗∗∗∗*^, ziprasidone^*∗∗*^)
Phenothiazines
Tricyclic antidepressants (amitriptyline^*∗∗*^, desipramine^*∗∗*^, clomipramine^*∗∗∗∗*^, nortriptyline^*∗*^)
Opiates (methadone, morphine, etc.)
Chlordiazepoxide
Amphetamines
Diazepam
Chlorpromazine
SSRIs (citalopram, fluoxetine, fluvoxamine, paroxetine, sertraline)
Other antidepressants (bupropion, venlafaxine, mirtazapine, nefazodone, trazodone)

*Hormones*
Estrogen
Oral-steroid contraceptives
Thyrotropin-releasing hormone
*Antihypertensives*
α-Methyldopa
Reserpine
Verapamil

*Dopamine receptor antagonists*
Metoclopramide

*Antiemetics*
Sulpiride
Promazine
Perphenazine
Metoclopramide^*∗∗∗∗*^
Domperidone (not available in United States)^*∗∗∗∗*^
Prochlorperazine^*∗∗*^

*Others*
Cimetidine
Cyproheptadine
Protease inhibitors

*Inhibitors*
l-Dopa
Dopamine
Bromocriptine
Pergolide
Cabergoline
Depot bromocriptine

Frequency of increase to abnormal prolactin levels with chronic use: ^*∗∗∗∗*^high >50 percent; ^*∗∗∗*^moderate: 25 to 50 percent; ^*∗∗*^low <25 percent; ^*∗*^none or low: case reports. Effect may be dose-dependent.

## References

[1] Melmed J. J. (2014). Anterior pituitary: physiology of pituitary hormones. *Harrison’s Principles of Internal Medicine*.

[2] Ra L. (2016). Hyperprolactinemia, galactorrhea, and pituitary adenomas: etiology, differential diagnosis, natural history, and management. *Comprehensive Gynecology*.

[3] Marc A., Fritz L. S. (2011). Hyperprolactinemia. *Clinical Gynecologic Endocrinology and Infertility*.

[4] Ibanez-Costa A., Luque R. M., Castano J. P. (2017). Cortistatin: a new link between the growth hormone/prolactin axis, stress, and metabolism. *Growth Hormone and IGF Research*.

[5] Glezer A., Bronstein M., De Groot L. J. (2000). Hyperprolactinemia. *Endotext*.

[6] Shelly S., Boaz M., Orbach H. (2012). Prolactin and autoimmunity. *Autoimmunity Reviews*.

[7] Bole-Feysot C., Goffin V., Edery M., Binart N., Kelly P. A. (1998). Prolactin (PRL) and its receptor: actions, signal transduction pathways and phenotypes observed in PRL receptor knockout mice. *Endocrine Reviews*.

[8] Vergani G., Mayerhofer A., Bartke A. (1994). Acute effects of rat growth hormone (GH), human GH and prolactin on proliferating rat liver cells in vitro: a study of mitotic behaviour and ultrastructural alterations. *Tissue and Cell*.

[9] Muller E., Dowling R. H. (1981). Prolactin and the small intestine. Effect of hyperprolactinaemia on mucosal structure in the rat. *Gut*.

[10] Sauro M. D., Zorn N. E. (1991). Prolactin induces proliferation of vascular smooth muscle cells through a protein kinase C-dependent mechanism. *Journal of Cellular Physiology*.

[11] Nielsen J. H., Møldrup A., Billestrup N. (1992). The role of growth hormone and prolactin in beta cell growth and regeneration. *Advances in Experimental Medicine and Biology*.

[12] Ochoa-Amaya J. E., Malucelli B. E., Cruz-Casallas P. E. (2010). Acute and chronic stress and the inflammatory response in hyperprolactinemic rats. *Neuroimmunomodulation*.

[13] Ruiz-Herrera X., de los Ríos E. A., Díaz J. M. (2017). Prolactin promotes adipose tissue fitness and insulin sensitivity in obese males. *Endocrinology*.

[14] Melmed J. J. (2014). Anterior pituitary tumor syndromes. *Harrison’s Principles of Internal Medicine*.

[15] Palubska S., Godlewska A. A., Winkler I. (2017). Hyperprolactinaemia–a problem in patients from the reproductive period to the menopause. *Menopausal Review*.

[16] Ittig S., Studerus E., Heitz U. (2017). Sex differences in prolactin levels in emerging psychosis: indication for enhanced stress reactivity in women. *Schizophrenia Research*.

[17] Muneyyirci-Delale O., Goldstein D., Reyes F. I. (1989). Diagnosis of stress-related hyperprolactinemia. Evaluation of the hyperprolactinemia rest test. *New York State Journal of Medicine*.

[18] Briet C., Saraval M., Loric S., Topolinski-Duyme H., Fendri S., Desailloud R. (2007). The use of intravenous catheterisation with a rest period is useful for determination of plasma cortisol levels but not plasma prolactin levels. *Annales d’Endocrinologie*.

[19] Me M. (2002). Prolactinoma. *The Pituitary*.

[20] Newey P. J., Gorvin C. M., Thakker R. V. (2014). Mutant prolactin receptor and familial hyperprolactinemia. *New England Journal of Medicine*.

[21] Shoupe D. (1997). Mishell’s textbook of infertility, contraception and reproductive endocrinology. *HDA Treatment*.

[22] Coker F., Taylor D. (2010). Antidepressant-induced hyperprolactinaemia: incidence, mechanisms and management. *CNS Drugs*.

[23] Molitch M. E. (2005). Medication-induced hyperprolactinemia. *Mayo Clinic Proceedings*.

[24] Molitch M. E. (2008). Drugs and prolactin. *Pituitary*.

[25] Yang P. C., Jury J., Söderholm J. D., Sherman P. M., McKay D. M., Perdue M. H. (2006). Chronic psychological stress in rats induces intestinal sensitization to luminal antigens. *American Journal of Pathology*.

[26] Akiyama H., Amano H., Bienenstock J. (2005). Rat tracheal epithelial responses to water avoidance stress. *Journal of Allergy and Clinical Immunology*.

[27] Sliwa K., Fett J., Elkayam U. (2006). Peripartum cardiomyopathy. *Lancet*.

[28] Song J., Wang M., Chen X. (2016). Prolactin mediates effects of chronic psychological stress on induction of fibrofatty cells in the heart. *American Journal of Translational Research*.

[29] Jaroenporn S., Nagaoka K., Kasahara C., Ohta R., Watanabe G., Taya K. (2007). Physiological roles of prolactin in the adrenocortical response to acute restraint stress. *Endocrine Journal*.

[30] Weber R. F., Calogero A. E. (1991). Prolactin stimulates rat hypothalamic corticotropin-releasing hormone and pituitary adrenocorticotropin secretion in vitro. *Neuroendocrinology*.

[31] Kooy A., de Greef W. J., Vreeburg J. T. M. (1990). Evidence for the involvement of corticotropin-releasing factor in the inhibition of gonadotropin release induced by hyperprolactinemia. *Neuroendocrinology*.

[32] Higuchi K., Nawata H., Maki T., Higashizima M., Kato K. I., Ibayashi H. (1984). Prolactin has a direct effect on adrenal androgen secretion. *Journal of Clinical Endocrinology and Metabolism*.

[33] Glasow A., Breidert M., Haidan A., Anderegg U., Kelly P. A., Bornstein S. R. (1996). Functional aspects of the effect of prolactin (PRL) on adrenal steroidogenesis and distribution of the PRL receptor in the human adrenal gland. *Journal of Clinical Endocrinology and Metabolism*.

[34] Chang L. L., Lo M. J., Kan S. F. (1999). Direct effects of prolactin on corticosterone release by zona fasciculata-reticularis cells from male rats. *Journal of Cellular Biochemistry*.

[35] Sonino N., Navarrini C., Ruini C., Fallo F., Boscaro M., Fava G. (2004). Life events in the pathogenesis of hyperprolactinemia. *European Journal of Endocrinology*.

[36] Machado R. B., Rocha M. R., Suchecki D. (2017). Brain prolactin is involved in stress-induced REM sleep rebound. *Hormones and Behavior*.

[37] Fujikawa T., Soya H., Tamashiro K. L. K. (2004). Prolactin prevents acute stress-induced hypocalcemia and ulcerogenesis by acting in the brain of rat. *Endocrinology*.

[38] Kirk S. E., Xie T. Y., Steyn F. J., Grattan D. R., Bunn S. J. (2017). Restraint stress increases prolactin-mediated phosphorylation of signal transducer and activator of transcription 5 in the hypothalamus and adrenal cortex in the male mouse. *Journal of Neuroendocrinology*.

[39] Perry B. I., Goldring K. J., Menon S. J. (2016). Prolactin monitoring in the acute psychiatry setting. *Psychiatry Research*.

[40] Riecher-Rossler A., Rybakowski J. K., Pflueger M. O. (2013). Hyperprolactinemia in antipsychotic-naive patients with first-episode psychosis. *Psychological Medicine*.

[41] Nunes M. C., Sobrinho L. G., Calhaz-Jorge C., Santos M. A., Mauricio J. C., Sousa M. F. (1980). Psychosomatic factors in patients with hyperprolactinemia and/or galactorrhea. *Obstetrics and Gynecology*.

[42] Sobrinho L. G. (2003). Prolactin, psychological stress and environment in humans: adaptation and maladaptation. *Pituitary*.

[43] Bo Q. J., Wang Z. M., Li X. B., Ma X., Wang C. Y., de Leon J. (2016). Adjunctive metformin for antipsychotic-induced hyperprolactinemia: a systematic review. *Psychiatry Research*.

[44] Krysiak R., Szkrobka W., Okopien B. (2017). Alternative treatment strategies in women poorly tolerating moderate doses of bromocriptine. *Experimental and Clinical Endocrinology and Diabetes*.

[45] Pompili M., Serafini G., Palermo M. (2013). Hypothalamic pituitary adrenal axis and prolactin abnormalities in suicidal behavior. *CNS and Neurological Disorders-Drug Targets*.

